# Effect of fixed orthodontic appliances on salivary properties

**DOI:** 10.1186/2196-1042-14-13

**Published:** 2013-06-18

**Authors:** Giulio Alessandri Bonetti, Serena Incerti Parenti, Giulia Garulli, Maria Rosaria Gatto, Luigi Checchi

**Affiliations:** Department of Orthodontics, University of Bologna, via San Vitale 59, Bologna, 40125 Italy; Department of Periodontology, University of Bologna, via San Vitale 59, Bologna, 40125 Italy

## Abstract

**Background:**

So far, a few studies have tried to investigate the relationship between the placement of fixed orthodontic appliances and the change of nonmicrobial salivary properties, mostly with conflicting outcomes and short-term assessment (up to 6 months from bracket placement). The purpose of this study was to evaluate the salivary flow rate, pH and buffer capacity prior to the beginning of therapy and after 1 year from bracket placement using a simple and commercially available chairside saliva check kit.

**Methods:**

The study population consisted of 20 healthy patients (mean age, 16.5 ± 4 years) scheduled for fixed orthodontic treatment. Salivary samples were taken just before bracket bonding (T0; baseline assessment) and after 1 year of treatment (T1; half-treatment assessment) using the GC Saliva-Check Kit (GC Corp., Leuven, Belgium).

**Results:**

No statistically significant difference was detected between T0 and T1 for the salivary parameters examined in the present study.

**Conclusions:**

Under the conditions of this study, the placement of fixed orthodontic appliances did not change the salivary pH, buffer capacity and flow rate after 1 year of treatment if compared with the baseline assessment.

## Background

The quality (defined as salivary protein content, viscosity, pH and buffer capacity) and the quantity of saliva (mostly related to flow rate) play a crucial role in the equilibrium between demineralization and remineralization of enamel in a cariogenic environment [1]. Specific changes, such as increased pH, buffer capacity and flow rate, may contribute to decreased susceptibility to dental caries [2, 3].

All these salivary properties become of utmost importance during orthodontic treatment with fixed appliances, when an increased chance of plaque retention and a greater difficulty in optimal oral hygiene maintenance are thought to predispose to enamel demineralization and white spot formation [4, 5]. There is still no consensus on the way the quality and the quantity of saliva change during orthodontic treatment [6]. So far, investigations have been confined to the first 6 months from the placement of fixed appliances, and no data are yet available in the long term [6–11].

The aim of this prospective study was, therefore, to evaluate the salivary pH, buffer capacity and flow rate at the beginning of orthodontic treatment (i.e. patients without fixed appliances) and after 1 year (i.e. patients with fixed appliances) by using a simple and commercially available chairside saliva check kit, under the null hypothesis that there was no significant difference between baseline and 1 year.

## Methods

In the present study, patients scheduled for fixed orthodontic treatment at the Department of Orthodontics, University of Bologna, Italy, were analyzed. The study protocol was approved by the local Institutional Review Board. The study was carried out in accordance with the ethical standards set forth in the 1964 Declaration of Helsinki, and informed consent was obtained from all participants prior to their enrollment.

Sample size was computed by saliva whole flow rate (SWFR), pH and buffer capacity, nonmicrobial salivary parameters that have been reported to significantly change after the placement of fixed orthodontic appliances [6]. A pilot study was carried out on a sample of 12 subjects [12]; the mean difference between baseline and 1 year was 0.1 ml/min for SWFR, 1 unit for pH and 0.6 unit for buffer capacity. By hypothesizing a mean difference of 0.1 unit with a standard deviation (SD) of 0.14, with a power of 80% and *α* = 0.05, a minimum number of 20 subjects was required for the study. Thus, 20 patients (11 males and 9 females; mean age, 16.5 ± 4 years) were selected from the Department of Orthodontics. Inclusion criteria were (1) good general health and (2) permanent dentition stage. Exclusion criteria were (1) patients taking any medication altering the salivary flow rate, (2) pregnancy, (3) smoking habit, (4) poor oral hygiene, (5) active caries or periodontal disease and (6) previous orthodontic treatment.

All subjects were treated with a straight wire technique using MBT Victory Series 0.022-inch-slot brackets (3 M Unitek, Monrovia, California, USA) on both the maxillary and the mandibular arches. Before the beginning of treatment, all patients underwent meticulous professional oral hygiene at the Department of Periodontology, University of Bologna, Italy; no clinical signs of unhealthy gingival conditions (probing pocket depths exceeding 3 mm and bleeding on probing) were present at that time. Oral hygiene instructions were given after fixed appliance placement, followed by proper reinforcement on each orthodontic adjustment appointment. Every 3 months during active orthodontic treatment, all subjects underwent professional oral hygiene maintenance; more frequent hygienic check-ups were scheduled in the presence of clinical signs of unhealthy gingival conditions.

### Salivary analysis

Salivary samples were taken between 9 a.m. and 12 noon, at least 2 h after meals and oral hygiene procedures in order to minimize the effects of diurnal variability in salivary composition [13]. Levels of labial hydration of unstimulated saliva (unstimulated flow rate, UFR), the resting pH, the volume of stimulated saliva (SWFR) and its buffer capacity were determined using the GC Saliva-Check Kit (GC Corp., Leuven, Belgium) [13]. For UFR, the lower lip was dried, and the time(s) taken for a salivary droplet to form was recorded. After the subjects were asked to pool and expectorate their saliva into a collection cup, a pH strip was immersed into the saliva sample for 10 s, and the colour change was used to estimate the resting pH according to the scale provided by the manufacturer. To stimulate saliva, the subjects were asked to chew a piece of paraffin wax, and saliva was collected for 5 min in a measuring cup with 1-ml gradation marks; the SWFR (ml/min) was calculated by dividing the amount of expectorated saliva by 5 min. One drop from the saliva sample was, finally, dispensed on a buffering strip using a pipette, left in place for 5 min, and the buffer capacity was recorded according to the scale provided by the manufacturer.

Salivary samples were taken from each patient immediately before the placement of fixed orthodontic appliances (T0; baseline assessment) and after 1 year (T1; half-treatment assessment). All procedures were performed by one operator (S.I.P.) with experience of over 50 salivary tests at the time of the study.

### Reliability

In order to test the intra-observer reliability, all the patients were re-examined by the same evaluator (S.I.P.) who recorded UFR, pH, SWFR and buffer capacity twice on the same day.

### Statistical analysis

The Shapiro-Wilk test was applied to verify the normality of distribution of the examined variables on the whole sample and, separately, for males and females. The data relative to UFR, SWFR and pH were not normally distributed; thus, median, interquartile range and SD of the median were used for descriptive purposes. Mann-Whitney and *U* tests were used to compare the data between the sexes; the Wilcoxon test for paired samples was used to investigate possible differences between T0 and T1. Since the data relative to the buffer capacity were consistent with a Gaussian distribution, arithmetic mean, range and SD of the mean were used for descriptive purposes. The *t* test for independent samples was applied for the comparison between the sexes, while the comparison between T0 and T1 was carried out with the *t* test for paired sample.

Intra-observer agreement was evaluated by Cronbach's alpha and intra-class correlation coefficient (ICC).

The level of significance was set at 0.05. Statistical analyses were performed using the statistical software SPSS for Windows (version 16.0, SPSS Inc., Chicago, IL, USA).

## Results

Since no statistically significant differences were found between males and females, data were combined for sex. No statistically significant difference existed between T0 and T1 for the nonmicrobial salivary properties examined in this study, except for a tendency to decrease between the two time points for buffer capacity (*p* = 0.05; Table 1).

**Table 1:** **Data for salivary unstimulated flow rate (UFR), resting pH, saliva whole flow rate (SWFR) and buffer capacity before (T0; baseline assessment) and 1 year after placement of fixed orthodontic appliances (T1; half-treatment assessment)**

		UFR (s)		pH		SWFR (ml/min)		Buffer capacity	
*n* = 20	Time points	T0	T1	T0	T1	T0	T1	T0	T1
	Median	37.0	37.5	6.8	6.9	1.4	1.4	7.4 (2.04)^a^	7.15 (2.23)^a^
	Interquartile range	17.5	39.25	1	1.15	0.83	0.93	8^b^	9^b^

^a^Mean (standard deviation); ^b^range.

Reliability analysis demonstrated an excellent intra-observer agreement for each salivary parameter (UFR:Cronbach's alpha = 0.997, ICC = 0.994; pH:Cronbach's alpha = 0.975, ICC = 0.952; SWFR:Cronbach's alpha = 0.979, ICC = 0.959; buffer capacity:Cronbach's alpha = 0.909, ICC = 0.833).

## Discussion

Many studies have been conducted to determine the changes of microbial environment in patients undergoing fixed orthodontic treatment [14–20]. On the other hand, a few previous reports have tried to investigate the relationship between fixed orthodontic appliances and the changes of nonmicrobial salivary properties, mostly with conflicting outcomes and short-term assessment (up to 6 months from bracket placement) [6–10].

Chang et al. [7] concluded that stimulated salivary flow rate, pH and buffer capacity significantly increase after 3 months of active orthodontic treatment. Similar results were described by Lara-Carrillo et al. [9] at 1 month from bracket placement. Peros et al. [6] found that salivary pH and stimulated flow rate significantly increase after, respectively, 12 and 18 weeks of fixed orthodontic treatment while buffer capacity remains almost unchanged after 18 weeks if compared to the baseline assessment. It arises from these data that an increase in the nonmicrobial salivary properties mentioned above can generally be detected in the early period from bracket placement [6, 7, 9]. Such a modification can be considered as a physiological response to the mechanical stimulation resulting from the presence of the fixed orthodontic appliances, as a result of a disturbed intra-oral homeostasis [9]. According to some authors [3, 8, 21–23], the increase in flow rate, pH and buffer capacity can have a sort of protective effect against the development of caries.

Since it has been suggested that a different period of evaluation should have been undertaken in order to determine the salivary conditions in the longer term [24], we decided to analyze the secretion time of unstimulated saliva, the resting pH, the volume of stimulated saliva and its buffer capacity after 1 year from bracket placement, a duration that was chosen as a sort of half orthodontic treatment assessment. It was our hypothesis that those nonmicrobial salivary parameters might have adjusted to the placement of fixed orthodontic appliances, thus returning towards the values observed prior to treatment with a sort of ‘adaptative behaviour’ occurring in the long term. Salivary analyses were carried out by one experienced and reliable operator using a simple and commercially available chairside saliva check kit (GC Corp., Leuven, Belgium), already employed for clinical investigations [9, 13, 23].

No significant difference was noted in stimulated flow rate, pH at rest and buffer capacity during the 1-year observation period. It was only for buffer capacity that a tendency towards reduction could be detected between the two observations but with no significance from a statistical perspective. These results are in contrast with previous findings reporting a general increase in the earlier period of treatment [6, 7, 9] but are somewhat similar to those by Sanpei et al. [25], reporting no significant differences in the scores of those nonmicrobial salivary parameters. However, direct comparison with the current study is inappropriate due to the differences in the number of brackets. Our findings are in agreement with those by Peros et al. [6], reporting the buffer capacity to remain almost unchanged after 18 weeks of treatment if compared to the baseline assessment, with a tendency towards reduction.

The unstimulated salivary flow rate remained statistically unchanged during the duration of the study in agreement with the study by Li et al. [10], who reported that salivary parameter being statistically unchanged after 6 months of fixed orthodontic treatment.

Since it is well known that the placement of a foreign body into the oral cavity initially causes a stimulation which increases the salivary flow [26, 27], it can be speculated that some kind of stimulus adaptation occurs in the long term. Thus, the initial increase in salivary pH, buffer capacity and flow rate of the first months of orthodontic treatment will probably decrease towards the baseline value up to 1 year from bracket placement. The clinical significance would be that, in the long term, there is no physiological change in salivary flow rate, pH and buffer capacity that could influence the risk of caries development during fixed orthodontic treatment. This should encourage the patient to initiate changes in oral hygiene and dietary habits and the dentist to plan appropriate prevention program after the placement of fixed orthodontic appliances in order to reduce the risk of caries development.

## Conclusions

Under the conditions of this study, the null hypothesis could not be rejected because no significant difference was detected in the salivary pH, buffer capacity and flow rate between orthodontic treatment beginning (i.e. patients without fixed appliances) and 1 year of treatment (i.e. patients with fixed appliances) by using a simple and commercially available chairside saliva check kit. These findings seem not to support the idea that changes in those nonmicrobial salivary properties are induced by fixed appliance placement, thus influencing the risk of caries development and confirm the importance of nutritional factors and proper oral hygiene maintenance in patients undergoing fixed orthodontic treatment.
